# Using the Health Belief Model to assess COVID-19 perceptions and behaviours among a group of Egyptian adults: a cross-sectional study

**DOI:** 10.1186/s12889-023-16513-x

**Published:** 2023-08-25

**Authors:** Dina N. K. Boulos, Azza Mohammed Hassan

**Affiliations:** 1 Department of Public Health, Faculty of Medicine, Ain Shams University, Cairo, Egypt; 2 Department of Public Health and Community Medicine, School of Medicine, Newgiza University, Giza, Egypt

**Keywords:** COVID-19, Health Belief Model, Preventive behaviour

## Abstract

**Background:**

It is crucial to study the public’s perceptions and behaviour during a pandemic as this will be the driving force for practicing recommended precautions. The current study aimed to identify perceptions of a group of Egyptian adults to COVID-19 using the Health Belief Model (HBM), to measure self-reported practice of preventive behaviours and to identify influencing factors.

**Methods:**

Cross sectional study was used, including Egyptian adults aged 18 + years. A structured anonymous online questionnaire was used including: a demographic section, the modified MERS- CoV Health Belief Model scale after addition of questions related to COVID-19 and questions on preventive behaviours to COVID-19.

**Results:**

Of the 532 study participants, 28.6% were males, age ranges (18 to 74 years). There was a statistically significant positive correlation between total practice score and all COVID-19 Health Belief Model constructs total scores except for perceived barriers score showing negative correlation (*P* value < 0.05). Linear regression analysis showed that older age, male gender and living inside Cairo were associated with lower practice score (*P* value < 0.01).

**Conclusions:**

Increased perceived susceptibility, perceived benefits, cues to action and perceived self-efficacy scores were associated with higher practice score in the current study. Additionally, results revealed that social media and websites can play an important role in shaping risk perception in the community. Stressing risk perception and efficacy beliefs prevention message can drive people to practice preventive behaviors.

**Supplementary Information:**

The online version contains supplementary material available at 10.1186/s12889-023-16513-x.

## Background

At the end of January 2020, the World Health Organization (WHO) declared the outbreak of the coronavirus disease 2019 (COVID-19) to be a global health emergency [1]. In the case of the coronavirus disease 2019 (COVID-19) the way the general public behave will have an important impact on the course of (COVID-19) pandemic [2]. This pandemic presents a unique challenge to the global public health; responding and mitigating its effect needs public health emergency preparedness which represents a shared responsibility between the government and the public [3].

Due to the absence of specific antiviral drugs for COVID-19 and even with the availability of a vaccine, no single strategy can control the COVID-19 pandemic. Consequently, communities play a critical role in preventing the rapid spread of the virus [4, 5].

In order to prevent human to human transmission, communities are required to adopt certain recommended behaviours like physical distancing (remain at least 1–2 m away from others,) frequent hand washing and wearing face mask [3, 5–9].

Egypt recorded the first case of COVID-19 in mid-February 2020 [10]. During March until the end of April 2020 the COVID 19 cases didn’t exceed 200 cases per day [10]. Many preventive measures were taken by the Egyptian government to control COVID-19 spread such as closing all educational facilities, recreational and religious places as well as applying curfew between 8 p.m. and 6 a.m., closing all airports, reducing to half the number of public sector employees and providing a fund for 300,000 seasonal workers to encourage them to stay at home^.^ [11]. The government began loosening regulations by cutting curfew hours by the beginning of the holy month of Ramadan, which is probably what caused the observed increase in cases in May and June 2020. Cases reached 416 cases per million (0.4 per thousand) in June 2020. Additionally, at the end of May 2020 after Ramadan month, a new ministerial decree stating that every person should wear a facemask when entering all public institutions and public and private transportation was imposed [11]. The government loosened regulations a month later by limiting daily curfews to just four hours between 12 and 4 AM. Shops and places of worship could reopen, and cafes, restaurants, and hotels were permitted to operate at 25% of capacity [11]. However, some people may not adhere to these measures.

Preventive authorities in various nations must examine how the public perceives COVID-19 and develop communication strategies accordingly. Risk perception models have been proposed to help public health planners in understanding the causes of people's noncompliance with health behaviours and have been shown to be useful in understanding and predicting COVID-19 behaviours [12].

According to the Health Belief Model (HBM), the decision to practice a certain health related behaviour is affected by many factors: perceived susceptibility to the health condition, awareness of the impact of disease on their health (perceived severity), perceived benefits of practicing the required behaviour, and perceived barriers and costs as well as cues to action that are motivational factors affecting behavioural change. Examples include internal cues like personal symptoms as well as external cues like information and reminders about diseases. The last construct is perceived self-efficacy that is the belief in one's capacity to carry out an action [13].

Through information sufficiency, which refers to information that satisfies one's needs as well as information gathering ability, knowledge plays a key role in influencing behaviour. Additionally, choosing the best resource for COVID-19 information is crucial for designing risk communication strategies [14].

It is crucial to study the public’s perceptions and behaviour during a pandemic as this will be the driving force for practicing recommended precautionary behaviours in addition it will pave the way to plan a successful risk communication intervention to achieve the desired changes in community behaviour [14].

The current study was conducted to identify beliefs and perceptions of a group of Egyptian adults to COVID-19 using the Health Belief Model constructs and to measure self-reported practice of the main preventive behaviours to COVID-19 in the week preceding the survey as well as to identify factors associated with this practice.

## Methods

### Study type and population

A cross sectional study was used. Egyptian adults over 18 years of age were eligible to be included in the study. The electronic questionnaire was forwarded via the virtual networks in WhatsApp and Facebook groups. Individuals were asked to optionally complete it and forward it to their friends and colleagues. Time needed to fill the questionnaire was 8–10 min. The data collection took place over 90 days from June 2020 to August 2020 after the declaration of the first confirmed case at the peak stage of the epidemic in Egypt.

### Sample size

Using Epi Info 7 program for sample size calculation and assuming that the proportion of participants who perceive low susceptibility for COVID-19 infection is 50%, with margin of error = 5% and at confidence level of 95%, sample size of at least 384 persons would be needed. The number of persons who participated in the present study and completed the online survey was 532 persons.

### Study tool

A structured anonymous online questionnaire was used and it included four main sections:

1. Demographic section that included questions such as age, gender, highest level of education attained, occupation, monthly income, daily transportation system most frequently used, chronic diseases and self- perceived general health.
2. The modified MERS- CoV Health Belief Model scale was used after receiving authors’ approval and after addition of questions related to COVID-19. It included 26 questions (Additional file 1) [15]:
  Questions on perceived threat (5 questions for the perceived susceptibility and one question for the perceived severity of COVID-19).
  Five questions on perceived benefits and 7 questions on perceived barriers to practice the recommended preventive behaviors to COVID-19.
  Six questions on perceived self- efficacy and 2 questions on cues to action to practice the recommended preventive behaviours to COVID-19.

All construct questions of the health belief model were on a 5-point Likert scale (from strongly agree to strongly disagree), and scoring was from 1 to 5. Higher scores mean higher perceived threat, benefits, barriers, cues to action and self- efficacy.

3. Questions on preventive behaviours to COVID-19 practiced in the seven days preceding the survey; such as wearing facial masks, practicing hand hygiene and social distancing behaviours (e.g., reducing the use of public transport, avoiding crowded places and postponing or cancelling social events). Answers were on a 5-point Likert scale (from always to never), and scoring was from 1 (for never) to 5 (for always).
4. Three questions related to COVID-19 health information: meeting health information needs and being sufficient (responses were rated on a 3-point Likert scale: meets my needs completely, meets my needs to some extent, does not meet it). As well as was it easy or difficult to find the information needed (answers were rated on a 4-point Likert scale (from very easy to very difficult) and the favourite source for information regarding the COVID-19 virus.

Using Cronbach’s alpha overall reliability of the tool was (0.83). Cronbach’s alpha coefficients for each construct and practice are as follows: perceived susceptibility (0.70), perceived barriers (0.77), perceived benefits (0.81), cues to action (0.69), self-efficacy (0.73) and practice (0.85). Four experts in health education and public health were consulted to verify the content validity, and their opinions were used to make the necessary revisions and corrections to the questionnaire's language.

### Ethical consideration

Study Approval from the Ethical Review Committee at Ain Sham University was obtained prior to the study. All online questionnaires were anonymous with an introduction explaining the research aims and assuring confidentiality of data. As it was an online questionnaire participants were informed that their consent is implied by submitting the completed survey. Also, approval of the authors of the modified MERS- CoV scale was obtained.

### Data management

Analysis of data was done using SPSS program version 23. Quantitative data were presented as minimum, maximum, mean and standard deviation and qualitative data were presented as number and percentage. Student t test was used to compare quantitative data between two independent groups and One Way ANOVA test was used to compare more than two groups. Pearson’s correlation coefficient was used to measure correlation between different quantitative data. Linear regression analysis was used to measure independent effect of different variables on practice score. *P* value ≤ 0.05 was considered statistically significant.

## Results

Of the 532 study participants, 28.6% were males and 71.4% were females. Their ages ranged from 18 to 74 years with mean of 35.78 ± 12.26 years. About 26% of the participants were aged 35–44 years, followed by 25% who were 25–34 years old. More than half of participants (54.9%) had University or high Institute education and 35.5% had professional work. Most of participants lived inside Cairo (78.2%), the mean number of rooms in their houses was 3.24 ± 1.13 rooms, mean number of persons living with them was 3.66 ± 1.46 persons and more than half of participants (56.8%) used their private car for transportation. About 36% of participants had chronic disease; the most frequent chronic disease was lung disease or asthma (17.7%) (Table 1).

**Table 1:** Study participants’ characteristics (Total number = 532)

		**N**		**Percent**	
**Age (years)**	** < 25**	124		23.3	
	**25 – 34**	133		25.0	
	**35 – 44**	140		26.3	
	**45 – 54**	89		16.7	
	** > 55**	46		8.6	
**Gender**	**Male**	152		28.6	
	**Female**	380		71.4	
**Education level**	**Secondary education**	85		16.0	
	**Technical/Commercial Diploma**	40		7.5	
	**University/High Institute**	292		54.9	
	**Post graduate**	115		21.6	
**Job**	**Not working**	45		8.5	
	**Retired**	23		4.3	
	**Student**	114		21.4	
	**Housewife**	75		14.1	
	**Professional work**	189		35.5	
	**Administrative work**	86		16.2	
**Residence**	**Inside Cairo**	416		78.2	
	**Outside Cairo**	116		21.8	
**Commonly used transportation method**	**Public transport**	132		24.8	
	**Taxi/Uber**	74		13.9	
	**Private car**	302		56.8%	
	**Walking on foot**	24		4.5%	
**Presence of chronic disease**	**DM**	28		5.3%	
	**Hypertension**	80		15.0%	
	**Lung disease or asthma**	94		17.7%	
	**Autoimmune diseases**	33		6.2%	
	**Liver disease**	18		3.4%	
	**Kidney disease**	23		4.3%	
		**Min**	**Max**	**Mean**	**SD**
**Age in years**		18	74	35.78	12.26
**Number of rooms in house**		1	12	3.24	1.13
**Number of persons in house**		0	8	3.66	1.46

Most of study participants (75%) believed that their COVID-19 information was to some extent sufficient, 74.1% found it easy or very easy to obtain needed information and the most frequent favorable source for obtaining information was WHO web site (28.9%) (Table 2).

**Table 2:** Study participants perceived COVID-19 information sufficiency, gathering capacity and preferred source

		**N**	**Percent**
**Currently, I have** **COVID-19 information that meets my needs** **(Information sufficiency)**	**Completely**	93	17.5
	**To some extent**	399	75.0
	**Doesn’t meet my needs**	40	7.5
**Was it easy or difficult to obtain needed information about COVID-19?** **(Gathering capacity)**	**Very easy**	133	25.0
	**Easy**	261	49.1
	**Not easy nor hard**	95	17.9
	**Hard**	35	6.6
	**Very hard**	8	1.5
**The most favorable source for obtaining information about COVID-19?**	**Face book**	47	8.8
	**Web sites**	117	22.0
	**WHO web site**	154	28.9
	**MOHP web site**	91	17.1
	**TV/Radio**	33	6.2
	**HC providers**	78	14.7
	**Family and friends**	12	2.3

Results of COVID-19 Health Belief Model constructs showed that concerning perceived susceptibility 52.3% of respondents agreed that there is a high probability to catch COVID-19 infection. Perceived severity was observed in 60.5% of respondents who agreed that thinking of the probability of catching COVID-19 scares them. Concerning perceived benefits, the least agreed on (37.8%) was benefits of hand washing, followed by 70.1% for wearing mask outside home. As regards, perceived barriers only 26.5% of study participants disagreed that they don’t feel comfortable wearing mask to prevent catching COVID-19 and 44.7% disagreed that always wearing face mask when going out costs lot of money. High perceived self-efficacy was reported in washing hands and wearing face mask accounting for 90.0% and 87.2% respectively. Study participants answers to cues to action revealed that 67.7% searched for new information about COVID-19 to update their information about the disease and 74.8% reported that when they receive new information about COVID-19 from TV or radio or internet they stop and think about it (Table 3).

**Table 3:** COVID-19 Health Belief Model constructs

	**Strongly agree /Agree**		**Don't agree nor disagree**		**Strongly disagree/** **Disagree**	
	**N**	**%**	**N**	**%**	**N**	**%**
***Perceived susceptibility:***						
There is high probability I will catch COVID-19	278	52.3	220	41.4	34	6.4
I feel I will catch COVID-19 in the future	186	35.0	289	54.3	57	10.7
I feel my possibility of getting COVID-19 is higher than other people	120	22.6	243	45.7	169	31.8
My family members are at risk of catching COVID-19	278	52.3	191	35.9	63	11.8
I don't care about COVID-19 and I practice my usual daily activities	62	11.7	68	12.8	402	75.6
***Perceived severity:***						
Thinking of the probability of catching COVID-19 scares me	322	60.5	116	21.8	94	17.7
***Perceived barriers:***						
Regular hand washing with water and soap is not convenient for me	60	11.3	49	9.2	423	79.5
Regular hand washing with soap and water is time consuming	27	5.1	32	6.0	473	88.9
Preventive measures for COVID-19 are difficult to apply in daily life	141	26.5	74	13.9	317	59.6
Hand washing using soap and water or using alcohol cost me lot of money	139	26.1	91	17.1	302	56.8
I don’t feel comfortable wearing mask to prevent catching COVID-19	323	60.7	68	12.8	141	26.5
Always wearing mask when going out cost me lot of money	195	36.7	99	18.6	238	44.7
It is difficult to stay at home and not going out unless it is mandatory	201	37.8	84	15.8	247	46.4
***Perceived benefits:***						
If I wash my hands regularly with soap and water I will not catch COVID-19	201	37.8	225	42.3	106	19.9
When I follow ministry of health COVID-19 recommendations I will decrease my chances of catching the disease	439	82.5	75	14.1	18	3.4
When I follow ministry of health COVID-19 recommendations I will help to decrease dissemination of infection	482	90.6	39	7.3	11	2.1
When I wear mask outside home I will not catch COVID-19	373	70.1	126	23.7	33	6.2
If I stay home and not going out unless it is I will not catch COVID-19	426	80.1	75	14.1	31	5.8
***Cues to Action:***						
I search for new information about COVID-19 to update my information about the disease	360	67.7	89	16.7	83	15.6
When I receive new information about COVID-19 from TV or radio or internet I stop and think about it	398	74.8	84	15.8	50	9.4
***Perceived self-efficacy:***						
I am able to recognize symptoms of COVID-19	358	67.3	115	21.6	59	11.1
I am able to follow ministry of health COVID-19 preventive measures recommendations on a daily basis	417	78.4	83	15.6	32	6.0
I can wash my hands with soap and water regularly	479	90.0	38	7.1	15	2.8
I can obtain required medical care when I feel symptoms of COVID-19	213	40.0	181	34.0	138	25.9
I can wear mask when I go outside home	464	87.2	33	6.2	35	6.6
I can search for new information about COVID-19 by myself	423	79.5	71	13.3	38	7.1

Total scores for COVID-19 Health Belief Model constructs were ranging from 5–23 with mean of 12.77 ± 3.03 for perceived susceptibility score, 1–5 with mean of 3.52 ± 1.10 for perceived severity score, 8–35 with mean of 23.99 ± 4.78 for perceived barriers score, 5–25 with mean of 10.75 ± 2.87 for perceived benefits score, 2–10 with mean of 4.45 ± 1.65 for cues to action score and 6–26 with mean of 13.02 ± 3.28 for perceived self-efficacy score (Data not shown).

Asking about self -reported practice of preventive behaviours in the seven days preceding the survey showed that 45.7% of participants usually keep a distance of at least 1 m between them and others, 60% always don’t shake hands or kiss others, 66.7% always cover their mouth and nose with tissue or elbow when sneezing or coughing, 35.7% usually don’t touch their eyes, nose or mouth with hands, 66.4% always wear mask outside home, 54.5% always don’t go outside home unless mandatory, 65.4% always avoid crowded places, 73.3% always avoid using public transportation, 63% always postpone or cancel social events or gatherings and 56% always wash their hands regularly with soap and water or sanitizers for at least 20 s after handling money or using ATM or touching any surfaces or objects. Total practice score ranged from 10–50 with mean of 17.2 ± 6.16 (Table 4).

**Table 4:** Self -reported practice of preventive behaviours in the seven days preceding the survey

	**Always**		**Usually**		**Sometimes**		**Rarely**		**Never**	
	**N**	**%**	**N**	**%**	**N**	**%**	**N**	**%**	**N**	**%**
**I kept a distance of at least 1 m between me and others**	152	28.6	243	45.7	100	18.8	28	5.3	9	1.7
**I didn't shake hands or kissed others**	319	60.0	127	23.9	45	8.5	22	4.1	19	3.6
**I covered my mouth and nose with tissue or elbow when sneezing or coughing**	355	66.7	109	20.5	49	9.2	11	2.1	8	1.5
**I didn't touch my eyes, nose or mouth with my hands**	118	22.2	190	35.7	149	28.0	49	9.2	26	4.9
**I wore mask outside home**	353	66.4	124	23.3	38	7.1	6	1.1	11	2.1
**I didn’t go outside home unless mandatory**	290	54.5	154	28.9	65	12.2	14	2.6	9	1.7
**I avoided crowded places**	348	65.4	140	26.3	32	6.0	7	1.3	5	0.9
**I avoided using public transportation**	390	73.3	66	12.4	35	6.6	22	4.1	19	3.6
**I postponed or cancelled social events or gathering**	335	63.0	116	21.8	49	9.2	21	3.9	11	2.1
**I washed my hands regularly with soap and water or sanitizers for at least 20 s after handling money or using ATM or touching any surfaces or objects,……**	298	56.0	133	25.0	67	12.6	18	3.4	16	3.0

There was a statistically significant positive correlation between total practice score and all COVID-19 Health Belief Model constructs total scores except for perceived barriers score where the correlation was negative (*P* value < 0.05) (Table 5).

**Table 5:** Correlation between total practice score and COVID-19 Health Belief Model constructs total scores

	**Practice score**	
	**r**^**a**^	***P* value**
**Perceived susceptibility score**	0.23	< 0.001
**Perceived severity score**	0.13	0.004
**Perceived barriers score**	- 0.21	< 0.001
**Perceived benefits score**	0.37	< 0.001
**Cues to action score**	0.41	< 0.001
**Perceived self-efficacy score**	0.48	< 0.001

^a^Pearson’s correlation coefficient

Relation between practice score and participants’ characteristics showed that females had significantly lower practice score than males (*p* value < 0.001). Compared to young age group older age groups had significantly lower practice scores (*p* value < 0.05). Participants with a university or higher education had significantly lower practice scores than those with high school education (*p* value < 0.05). Participants living inside Cairo had significantly lower practice score than those living outside Cairo (*p* value < 0.05). Regarding occupation, retired persons had the lowest practice score while students had the highest score (*p* value < 0.05). Regarding transportation method, those who usually use taxi/Uber had the lowest practice scores while those walking on foot had the highest score (*p* value < 0.05). No significant relation was found between presence of chronic disease and practice score (*p* value > 0.05) (Table 6).

**Table 6:** Relation between practice score and participants’ characteristics

		**Practice**
**Gender**	**Male**	19.20 ± 7.09
	**Female**	16.28 ± 5.54
	***P* value**^**a**^	< 0.001
**Age (years)**	** < 25**	18.48 ± 6.61
	**25 -**	18.50 ± 7.26
	**35 -**	16.14 ± 5.43
	**45 -**	15.82 ± 4.69
	** > 55**	14.93 ± 4.13
	***P* value**^**b**^	< 0.001
**Education level**	**High school**	18.71 ± 6.77
	**University or higher**	16.63 ± 5.88
	***P* value**^**a**^	0.01
**Address**	**Inside Cairo**	16.67 ± 5.42
	**Outside Cairo**	18.72 ± 8.10
	***P* value**^**a**^	0.01
**Job**	**Not working**	18.07 ± 7.84
	**Retired**	14.70 ± 3.24
	**Student**	18.25 ± 6.44
	**Housewife**	16.75 ± 5.77
	**Professional work**	16.24 ± 5.55
	**Administrative work**	18.01 ± 6.59
	***P* value**^**b**^	0.01
**Usually used transport method**	**Public transport**	19.32 ± 7.18
	**Taxi/Uber**	15.74 ± 4.61
	**Private car**	16.14 ± 5.09
	**Walking on foot**	21.58 ± 10.32
	***P* value**^**b**^	< 0.001
**Chronic disease**	**Yes**	16.90 ± 5.77
	**No**	17.24 ± 6.37
	***P* value**^**a**^	0.53

^a^student t test

^b^One Way ANOVA test

Linear regression analysis was done to measure independent effect of different factors on practice score and showed that older age, male gender and living inside Cairo were associated with lower practice score while increased perceived susceptibility, increased cues to action and increased perceived self-efficacy scores were associated with higher practice score (*p* value < 0.01). The strongest predictor was perceived self-efficacy score as it has the highest standardized coefficient (0.194). (Table 7).

**Table 7:** Linear regression analysis for factors affecting practice score

	**Unstandardized Coefficients**		**Standardized Coefficients**	**T**	**Sig**	**95.0% Confidence Interval for B**	
	**B**	**Std. Error**	**Beta**			**Lower Bound**	**Upper Bound**
**(Constant)**	20.550	2.650		7.753	< 0.001	15.343	25.757
**Age (years)**	-.049	.021	-.098	-2.322	0.021	-.091	-.008
**Male gender**	-1.886	.555	-.137	-3.397	0.001	-2.976	-.795
**Living inside Cairo**	-1.379	.594	-.092	-2.320	0.021	-2.546	-.211
**Presence of chronic disease**	.309	.542	.024	.571	0.568	-.755	1.374
**Perceived susceptibility score**	.288	.084	.147	3.416	0.001	.122	.453
**Perceived severity score**	-.056	.246	-.010	-.226	0.821	-.539	.428
**Perceived barriers score**	-.023	.052	-.019	-.435	0.664	-.125	.080
**Perceived benefits score**	.147	.106	.070	1.384	0.167	-.062	.355
**Cues to action score**	.584	.183	.156	3.193	0.001	.225	.944
**Perceived self-efficacy score**	.363	.102	.194	3.559	< 0.001	.162	.563

## Discussion

COVID-19 pandemic has intensely affected peoples' lives and the required preventive behaviours such as the complete or partial lock-down, curfew hours, social distancing, wearing face mask, frequent hand washing have impacted the daily routine of individuals. The current study was conducted to investigate the factors affecting the practice of the recommended preventive behaviours to COVID-19 by applying the HBM constructs to assess the relationship between perceptions regarding COVID-19 as an emerging disease and the precautionary behaviours practice of the study participants. The present study results also highlighted the sociodemographic factors related to COVID-19 perceived susceptibility, perceived severity, perceived benefits, and perceived barriers and cues to action and practice score.

The most frequently reported preventive behaviours by the study participants in the seven days preceding the survey were always avoiding using public transportation (73.3%.), covering mouth and nose with tissue or elbow when sneezing or coughing and wearing mask outside home accounting for 66.7% and 66.4% respectively. Always washing hands regularly with soap and water or sanitizers for at least 20 s after handling money or using ATM or touching any surfaces or objects was less frequently reported (56%). These percentages are similar to the results of a study in Pakistan in which wearing face mask when outside was reported by 71% while washing hands and using sanitizers was practiced by 60% of the study participants [4]. The results of a survey of Chinese general population in Wuhan and Shanghai revealed that 86.7% often wear a face mask when outside. The much higher engagement in the precautionary behaviour reported in the latter study may be due to the fact that China and especially Wuhan is the epicentre of the pandemic and consequently people may be more anxious to follow the preventive behaviours [16]. Inversely, always wearing a face mask when outside was only reported by 25.1% of asymptomatic Latino population in the United States of America [17]. In addition, the most frequently reported precautionary behaviour in many studies from Asia is washing hands and using sanitizers [4, 18]. The latter behaviour was among the less frequently reported behaviours among the current study participants. The difference may be due to the question itself as in the present study, the question specified the duration of hand washing and the situations as well as answers were graded on a Likert scale from always to never, not a dichotomous yes or no answer consequently may be fewer positive responses were reported. Also, it may be difficult to find a source of clean water outside home and sanitizers are not commonly used by the Egyptian community as they are expensive.

It is crucial to understand the key elements that influence individuals’ compliance to the required practice of preventive behaviours to be able to tailor future risk communication interventions. The current study showed some sociodemographic factors that impact perceived susceptibility, severity, benefits and barriers as well as cues to action and perceived self-efficacy. Linear regression analysis revealed that older age, male gender, living inside Cairo and high perceived barrier score were independent variables for lower practice score of preventive behaviours. A better practice of precautionary behaviour among women was also observed in other studies [19, 20]. Lau et al. showed that, in the case of the 2009 H1N1 epidemic in Hong Kong, women followed precautionary behaviours more often than men [20]. Furthermore, in the present study older age was an independent statistically significant variable for low practice score this finding agrees with Azlan et al. study among the Malaysian public that showed older age group above the age of 50 were less likely to avoid crowded places or wear face masks [21].

Similarly, an online study that assessed the level of preparedness for disasters caused by COVID-19 in Serbia revealed that younger study participants reported the highest level of restriction on movement as a preventive measure [22]. Inversely, an online survey in North America and Europe to determine barriers and facilitators to adherence to social distancing during COVID-19 showed that older study participants (> 45 years old) were more likely than younger individuals (18–24 years old) to adhere to social distancing measures by avoiding socializing in person and maintaining a safe distance (1–2 m) in public places [22].

In the current study bivariate analysis showed that perceived severity and perceived benefits increased significantly with age (*p* < 0.001, *p* = 0.002) respectively and contrariwise perceived self-efficacy, cues to action and practice score decreased significantly with age (*p* = 0.1, *p* < 0.001 and *p* < 0.001) respectively. This may be due to the fact that although there is high perception of severity there is less self-efficacy and cues to actions whether external as exposure to media, social media or internal so may be older age needs tailored interventions targeting them.

Then again, perceived barriers refer to an individual’s perception of obstacles to behavioural change [23]. examples of barriers include the costs, perceived risk of side effects and discomfort related to practicing specific preventive behaviour. Perceived benefits and perceived barriers are the opposite of each other. If the perceived benefit from a specific practice outweighs the perceived barrier, the individual may practice the preventive behaviour. In the current study perceived barriers were inversely related to the practice score of COVID-19 preventive behaviors. This agrees with the findings of a study from Iran in which increased perceived barriers and fatalistic beliefs significantly reduced the adherence to the recommended precautionary behaviours [23]. The main reported barriers in the above mentioned study were environmental barriers such as lack of availability of face masks, sanitizers and alcohol pads [24]. In the current study cost of wearing masks and using soap and water and sanitizers were perceived as barriers by 36.7% and 26.1% of study participants respectively.

In the present study increased perceived susceptibility, increased perceived benefits, increased cues to action and increased perceived self-efficacy scores were associated with higher practice score. These findings agree with Park et al., study results showing that study participants who perceived the severity of contracting H1N1 influenza reported washing their hands more frequently [25].

Additionally, the current study results suggest that social media and websites can play an important role in risk communication and shaping risk perception in the community as 76.8% of study participants reported WHO website, Other websites, MOHP website and Facebook as their most favourable source for obtaining information on COVID-19. The most favourable source was WHO website (28.9%) followed by other websites (22.0%), Ministry of Health and Population website (17.1%) and Facebook (8.8%). Similarly, a study from China revealed that 97.8% of study participants' main sources of pandemic information were social networking apps like WeChat [26]. The rising trend of using social media to access information has been observed in many studies and this may be due to increasing ease of access and appeal. Consequently, policymakers should carefully assess the quality of information transmitted through social networks during pandemics like the COVID-19 outbreak. Furthermore, a range of information sources should be employed when conveying vital health information to ensure that various populations have timely access to critical knowledge [27].

The findings of this study show the need of using HBM components as a framework during pandemics or epidemics to tailor interventions that can affect community-wide health-promoting behaviours, particularly interventions that target barriers and improve self-efficacy of vulnerable populations especially older age groups. The current study also demonstrated that older age, male gender living inside Cairo as well as perceived barriers were inverse variables significantly affecting practice. So, it's important to concentrate the efforts on the obstacles especially the ones associated as providing face masks and sanitizers at affordable cost as well as increasing awareness campaigns targeting older age group and Cairo residents.

Perceived illness risk and belief in the effectiveness of protective measures are the key factors influencing the adoption of precautionary behaviors during pandemic scenarios. According to the findings of this study, stressing risk perception and efficacy beliefs in the COVID-19 prevention message can drive people to engage in preventive actions. This agrees with Guidry et al. 2020 study that showed willingness to get COVID 19 was [positively predicted by high perceived susceptibility to COVID-19, high perceived benefits of the vaccine, low barriers to the vaccine, and scoring high on self-efficacy for getting the vaccine [28].

The present study was conducted in the early phase of pandemic further researches in subsequent waves can be highly beneficial in understanding community behaviour in pandemics. A framework for the modification of beliefs and adherence to COVID-19 preventive behaviour might be created by designing an educational intervention on the basis of the HBM especially by providing cues to action by way of showing various reminders via social media to prevent illness. The discovery of the obstacles and drivers of different behaviors could provide helpful points for the creation of communication strategies. This can be used to overcome COVID 19 vaccine hesitancy as well as immunizations reluctances and customise additional interventions. When creating initiatives and public health campaigns that promote vaccination it is important to take health beliefs into consideration. However, such campaigns should consider not just the HBM components but also geographic (country, area), socioeconomic status (income, education), and other demographic (age, gender, and employment) aspects that can affect a person's decision to get immunized or engage in any other healthy behavior.

### Limitations of the study

Due to the COVID- 19 limiting situation, data was collected using an electronic questionnaire forwarded via the virtual networks in WhatsApp and Facebook groups. As a result, convenient sampling was used consequently study results cannot be generalized to the Egyptian adult population. Furthermore, older adults and illiterate groups were not included in the study sample as they are less likely to use social media and internet and are not able or interested to fill such surveys.

## Conclusions

Increased perceived susceptibility, perceived benefits, cues to action and perceived self-efficacy scores were associated with higher practice score in the current study. Additionally, results revealed that social media and websites can play an important role in shaping risk perception in the community. Stressing risk perception and efficacy beliefs prevention message can drive people to practice preventive behaviors.

### Supplementary Information

**Additional file 1.**

## Data Availability

The authors have full control of all primary data and they agree to allow the journal to review their data upon request to the corresponding author.

## Abbreviations

WHO: World Health Organization
COVID-19: Coronavirus disease 2019
HBM: Health Belief Model
SD: Standard Deviation
