# Supplementary material for: Using the Health Belief Model to assess COVID-19 perceptions and behaviours among a group of Egyptian adults: a cross-sectional study
Source: BMC Public Health. 2023 Aug 25;23:1624. doi: 10.1186/s12889-023-16513-x (PMC10464151; doi:10.1186/s12889-023-16513-x)
Supplement: Supplementary file 1 — Additional file 1. [file 12889_2023_16513_MOESM1_ESM.docx]

**Appendix I**

**The Health Belief Model to Assess COVID-19 Perceptions and Behaviors among a group of Egyptian Adults**

This questionnaire is about health beliefs to assess cognition and behaviors specific to COVID-19. The community plays a critical role in preventing the rapid spread of the virus. It is important to study the public's interaction and behavior during the epidemic, as this will pave the way for planning successful health programs to achieve the desired changes in the behavior of society to face the threat of the epidemic.

We thank you for your kind cooperation and time, we assure you that data that will be collected will be treated with utmost confidentiality and will be used for this scientific research only.

**Section 1: Sociodemographic**

1**. Gender:** 1- Male 2- Female

2. **Age (in years):**

3**. Highest Certification Obtained:**

1- Technical/ Commercial Diploma 2- General Secondary 3- University/ Institute 4- Postgraduate Studies

4. **Residence:** 1- Inside Cairo 2- Outside Cairo

5. **Occupation:** 1- Housewife 2- Pension 3- I am not currently working 4- Student 5- Administrative work (secretarial or employee) 6- Professional (Doctor - Engineer - Accountant - Teacher ....) 7- Sales 9 - Armed Forces / Police

6. **Number of rooms in the house:**

7. **The number of people staying with you in the house:**

**8. Commonly used transportation method:**

1- Public means (metro - microbus - bus)

2- Taxi or Opera Karim or the like

3- A private car

4- walk

**9. Do you suffer from chronic diseases:**

1- Diabetes: 1- Yes 2- No

2- Heart and high blood pressure: 1- yes 2- no

3- Lung problems or asthma: 1- Yes 2- No

4- Immunological diseases: 1- Yes 2- No

5- Liver problems: 1- Yes 2- No

6- Kidney problems: 1- Yes 2- No

**Section 2: COVID-19 Health Belief Model constructs**

|  | **Strongly agree** | **Agree** | **Don't agree nor disagree** | **Disagree** | | **Strongly disagree/** |  |  |  |
| --- | --- | --- | --- | --- | --- | --- | --- | --- | --- |
| ***Perceived susceptibility:*** | | | | | | |  |  | ***Perceived susceptibility:*** |
| 10. There is high probability I will catch COVID-19 |  |  |  |  |  |  |  |  |  |
| 11. I feel I will catch COVID-19 in the future |  |  |  |  |  |  |  |  |  |
| 12. I feel my possibility of getting COVID-19 is higher than other people |  |  |  |  |  |  |  |  |  |
| 13. My family members are at risk of catching COVID-19 |  |  |  |  |  |  |  |  |  |
| 14. I don't care about COVID-19 and I practice my usual daily activities |  |  |  |  |  |  |  |  |  |
| ***Perceived severity:*** | | | | | | |  |  |  |
| 15. Thinking of the probability of catching COVID-19 scares me |  |  |  |  |  |  |  |  |  |
| 16. If I catch COVID-19 I will die |  |  |  |  |  |  |  |  |  |
| 17. If I catch COVID-19 other family members will get it |  |  |  |  |  |  |  |  |  |
| 18. COVID-19 is not a very dangerous disease |  |  |  |  |  |  |  |  |  |
| ***Perceived barriers:*** | | | | | | |  |  |  |
| 19. Regular hand washing with water and soap is not convenient for me |  |  |  |  |  |  |  |  |  |
| 20. Regular hand washing with soap and water is time consuming |  |  |  |  |  |  |  |  |  |
| 21. Preventive measures for COVID-19 are difficult to apply in daily life |  |  |  |  |  |  |  |  |  |
| 22. Hand washing using soap and water or using alcohol cost me lot of money |  |  |  |  |  |  |  |  |  |
| 23. I don’t feel comfortable wearing mask to prevent catching COVID-19 |  |  |  |  |  |  |  |  |  |
| 24. Always wearing mask when going out cost me lot of money |  |  |  |  |  |  |  |  |  |
| 25. It is difficult to stay at home and not going out unless it is mandatory |  |  |  |  |  |  |  |  |  |
| ***Perceived benefits:*** | | | | | | |  |  |  |
| 26. If I wash my hands regularly with soap and water I will not catch COVID-19 |  |  |  |  |  |  |  |  |  |
| 27.When I follow ministry of health COVID-19 recommendations I will decrease my chances of catching the disease |  |  |  |  |  |  |  |  |  |
| 28.When I follow ministry of health COVID-19 recommendations I will help to decrease dissemination of infection |  |  |  |  |  |  |  |  |  |
| 29.When I wear mask outside home I will not catch COVID-19 |  |  |  |  |  |  |  |  |  |
| 30.If I stay home and not going out unless it is I will not catch COVID-19 |  |  |  |  |  |  |  |  |  |

|  | **Strongly agree** | **Agree** | **Don't agree nor disagree** | **Disagree** | **Strongly disagree/** |
| --- | --- | --- | --- | --- | --- |
| ***Cues to Action:*** | | | | | |
| 31. I search for new information about COVID-19 to update my information about the disease |  |  |  |  |  |
| 32. When I receive new information about COVID-19 from TV or radio or internet I stop and think about it |  |  |  |  |  |
| ***Perceived self-efficacy:*** | | | | | |
| 33. I am able to recognize symptoms of COVID-19 |  |  |  |  |  |
| 34. I am able to follow ministry of health COVID-19 preventive measures recommendations on a daily basis |  |  |  |  |  |
| 35. I can wash my hands with soap and water regularly |  |  |  |  |  |
| 36. I can obtain required medical care when I feel symptoms of COVID-19 |  |  |  |  |  |
| 37. I can wear mask when I go outside home |  |  |  |  |  |
| 38. I can search for new information about COVID-19 by myself |  |  |  |  |  |

**Section 3: Self -reported practice of preventive behaviours**

|  | **Always** | | **Usually** | **Sometimes** | | **Rarely** | | | | | **Never** |  |
| --- | --- | --- | --- | --- | --- | --- | --- | --- | --- | --- | --- | --- |
| 39. During the preceding week I kept a distance of at least 1 meter between me and others |  |  | |  | |  | | |  | | |  |
| 40. During the preceding week I didn't shake hands or kissed others |  |  | |  | |  | |  | | | |  |
| 41. During the preceding week I covered my mouth and nose with tissue or elbow when sneezing or coughing |  |  | |  | |  | |  | | | |  |
| 42. During the preceding week I didn't touch my eyes, nose or mouth with my hands |  | |  | |  | |  | | |  | | |
| 43. During the preceding week I wore mask outside home |  | |  | |  | |  | | |  | |  |
| 44. During the preceding week I didn’t go outside home unless mandatory |  | |  | |  | |  | | |  | |  |
| 45. During the preceding week I avoided crowded places |  | |  | |  | |  | | |  | |  |
| 46. During the preceding week I avoided using public transportation |  | |  | |  | |  | | |  | |  |
| 47. During the preceding week I postponed or cancelled social events or gathering |  | |  | |  | |  | | |  | |  |
| 48. During the preceding week I washed my hands regularly with soap and water or sanitizers for at least 20 sec after handling money or using ATM or touching any surfaces or objects,…… |  | |  | |  | |  | | |  | |  |

**49. Currently, I have COVID-19 information that meets my needs**

1. Completely
2. To some extent
3. Doesn’t meet my needs

**50. Was it easy or difficult to obtain needed information about COVID-19?**

1. Very easy
2. Easy
3. Not easy nor hard
4. Hard
5. Very hard

**51. The most favorable source for obtaining information about COVID-19?**

- 1. Facebook
  2. Web sites
  3. WHO web site
  4. MOHP web site
  5. TV/Radio
  6. HC providers
  7. Family and friends
